# Neuroimaging in Primary Coenzyme-Q_10_-Deficiency Disorders

**DOI:** 10.3390/antiox12030718

**Published:** 2023-03-14

**Authors:** Juliane Münch, Jannik Prasuhn, Lucia Laugwitz, Cheuk-Wing Fung, Brian H.-Y. Chung, Marcello Bellusci, Ertan Mayatepek, Dirk Klee, Felix Distelmaier

**Affiliations:** 1 Department of General Pediatrics, Neonatology and Pediatric Cardiology, University Children’s Hospital, Medical Faculty, Heinrich Heine University Düsseldorf, Moorenstr. 5, 40225 Düsseldorf, Germany; 2 Institute of Neurogenetics, University of Lübeck, 23588 Lübeck, Germany; 3 Department of Neurology, University Medical Center Schleswig-Holstein, Campus Lübeck, 23562 Lübeck, Germany; 4 Center for Brain, Behavior, and Metabolism, University of Lübeck, 23562 Lübeck, Germany; 5 Department of Neuropediatrics, Developmental Neurology and Social Pediatrics, University of Tübingen, 72076 Tübingen, Germany; 6 Institute of Medical Genetics and Applied Genomics, University of Tübingen, 72076 Tübingen, Germany; 7 Department of Paediatrics and Adolescent Medicine, Li Ka Shing Faculty of Medicine Queen Mary Hospital, The University of Hong Kong, Hong Kong, China; 8 Reference Center for Inherited Metabolic Disorders, MetabERN Center “12 de Octubre” University Hospital, 28041 Madrid, Spain; 9 Mitochondrial & Neuromuscular Disorders Research Group, Instituto de Investigación Sanitaria “12 de Octubre” (imas12), 28041 Madrid, Spain; 10 Spanish Biomedical Research Networking Center in Rare Diseases (CIBERER), 28029 Madrid, Spain; 11 Department of Pediatric Radiology, Medical Faculty, Institute of Radiology, Heinrich-Heine-University, 40225 Düsseldorf, Germany

**Keywords:** ubiquinone, mitochondrial oxidative phosphorylation, neurodegeneration, multiple system atrophy, Leigh syndrome

## Abstract

Coenzyme Q_10_ (CoQ_10_) is an endogenously synthesized lipid molecule. It is best known for its role as a cofactor within the mitochondrial respiratory chain where it functions in electron transfer and ATP synthesis. However, there are many other cellular pathways that also depend on the CoQ_10_ supply (redox homeostasis, ferroptosis and sulfide oxidation). The CoQ_10_ biosynthesis pathway consists of several enzymes, which are encoded by the nuclear DNA. The majority of these enzymes are responsible for modifications of the CoQ-head group (benzoquinone ring). Only three enzymes (PDSS1, PDSS2 and COQ2) are required for assembly and attachment of the polyisoprenoid side chain. The head-modifying enzymes may assemble into resolvable domains, representing COQ complexes. During the last two decades, numerous inborn errors in CoQ_10_ biosynthesis enzymes have been identified. Thus far, 11 disease genes are known (*PDSS1*, *PDSS2*, *COQ2*, *COQ4*, *COQ5*, *COQ6*, *COQ7*, *COQ8A*, *COQ8B*, *COQ9* and *HPDL*). Disease onset is highly variable and ranges from the neonatal period to late adulthood. CoQ_10_ deficiency exerts detrimental effects on the nervous system. Potential consequences are neuronal death, neuroinflammation and cerebral gliosis. Clinical features include encephalopathy, regression, movement disorders, epilepsy and intellectual disability. Brain magnetic resonance imaging (MRI) is the most important tool for diagnostic evaluation of neurological damage in individuals with CoQ_10_ deficiency. However, due to the rarity of the different gene defects, information on disease manifestations within the central nervous system is scarce. This review aims to provide an overview of brain MRI patterns observed in primary CoQ_10_ biosynthesis disorders and to highlight disease-specific findings.

## 1. Introduction

**Figure 1:**
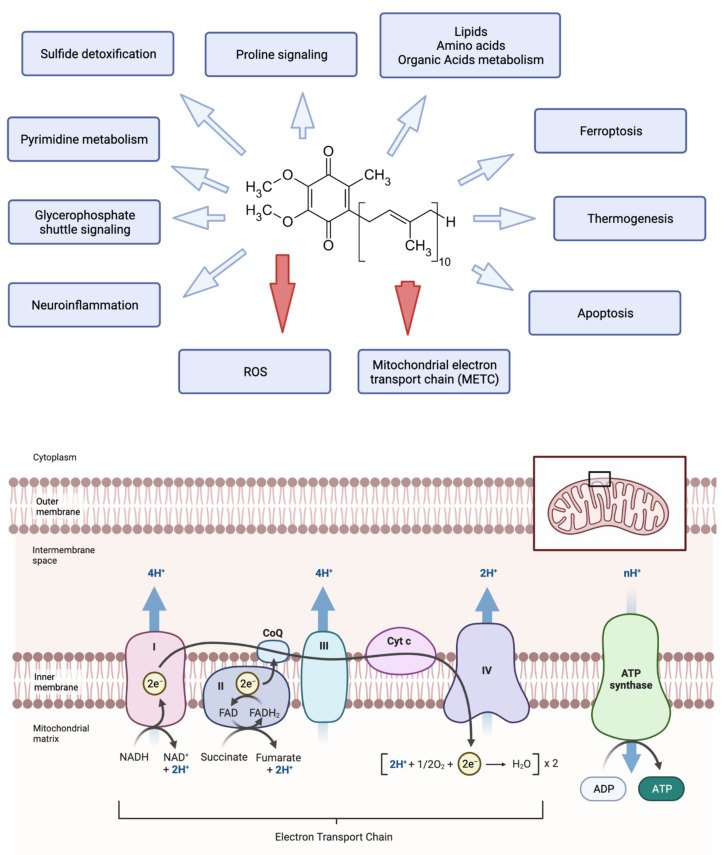
The multiple functions of CoQ_10_ in cell metabolism. CoQ_10_ is important for the function of numerous cellular pathways (upper panel). However, it is best known for its role in as an electron carrier within the mitochondrial respiratory chain (lower panel). In addition, it functions as a reactive oxygen species (ROS) scavenger that protects the cell against oxidative stress. Created with BioRender.com (accessed on 31 January 2023).

Coenzyme Q_10_ (ubiquinone) is a lipid molecule composed of a benzoquinone ring and a species-specific side chain. In humans, this side chain consists of 10 isoprene units [1]. CoQ_10_ is an essential cofactor in the mitochondrial oxidative phosphorylation (OXPHOS) system where it acts as an electron carrier between mitochondrial complex I, II and III (see Figure 1). Moreover, it functions as an antioxidant within the cell (see Figure 1) [2,3]. The requirements for CoQ_10_ are mainly supplied by endogenous de novo synthesis [4].

A distinction is made between primary (genetic) and secondary (alimentary) CoQ_10_ deficiency. The primary form affects the proteins that are directly involved in the synthesis of CoQ_10_ [5]. To date, disease-causing variants in *PDSS1*, *PDSS2*, *COQ2*, *COQ4*, *COQ5*, *COQ6*, *COQ7*, *COQ8A (ADCK3)*, *COQ8B (ADCK4)* and *COQ9* are known. Moreover, *HPDL* was recently identified as a gene involved in CoQ_10_ biosynthesis and human disease [6]. 

Clinically, CoQ_10_ deficiency disorders present with a broad spectrum of disease features and may manifest at any age. Most frequently, the brain, muscles and kidneys are affected. It is well-known that endogenous CoQ_10_ synthesis is crucial for neuronal development and function. ATP production via the mitochondrial OXPHOS system is essential for adequate energy supply, and in particular tissues with high metabolic demands are vulnerable to inherited defects affecting this pathway. 

Apart from bioenergetics, other crucial pathways within the nervous system are also regulated by CoQ_10_. This includes ferroptosis [7], sulfide oxidation [8], neuroinflammation [9] and redox homeostasis [10] (see Figure 1). Studies in mice with primary CoQ_10_ deficiency due to COQ9 knock-out (Coq9R239X) showed cerebral gliosis and spongiosis [9]. Moreover, COQ7 deficiency in mice induced a microglial metabolic reprogramming with subsequent neuronal cell death [11]. A neuronal cell model using para-aminobenzoic acid, a competitive inhibitor of the COQ2 enzyme, revealed a decrease in cellular ATP production and increased oxidative stress [12].

In humans, primary CoQ_10_ deficiency may affect the central as well as the peripheral nervous system. Brain pathology is mainly accessed via magnetic resonance imaging (MRI) and MR spectroscopy. Apart from single genetic defects (e.g. pathogenic variants in *COQ4* and *COQ8A*) information on neuroimaging abnormalities in primary CoQ_10_ deficiency is scarce. Therefore, the aim of this study was to explore the spectrum of CoQ_10_ deficiency-related brain MRI patterns.

## 2. Materials and Methods

During the time period 15 November until 10 December 2022, we searched PubMed for all reported cases of primary CoQ_10_ biosynthesis defects using the search terms “PDSS1”, “PDSS2”, “COQ2”, “COQ4”, “COQ5”, “COQ6”, “COQ7”, “COQ8A”, “ADCK3”, “COQ8B”, “ADCK4”, “COQ9”, ”Coenzyme Q10 deficiency” “primary coenzyme Q_10_ deficiency” and “coenzyme Q_10_”. We identified those publications in which brain imaging was described or demonstrated.

A total number of 150 publications were further screened, and finally 75 were incorporated into this review. The study was conducted in accordance with the Declaration of Helsinki. Regarding exemplary MRI images shown in this article, the families agreed to share medical information for scientific publication.

## 3. Results

### 3.1. PDSS1 Deficiency

*PDSS1* encodes for the prenyldiphosphate synthase, subunit 1 that elongates the prenyl sidechain of CoQ in the quinone biosynthesis pathway and was first described by Mollet et al. in 2007 [13]. Individuals with disease-causing variants in *PDSS1* typically present with deafness, optic atrophy and muscular hypotonia (primary coenzyme Q_10_ deficiency-2; COQ10D2; OMIM #614651). In addition, variable neurological features can be associated with the disease. There are only a few cases published in the literature. Brain MRI was performed in only four individuals [13,14,15,16]. 

**Figure 2:**
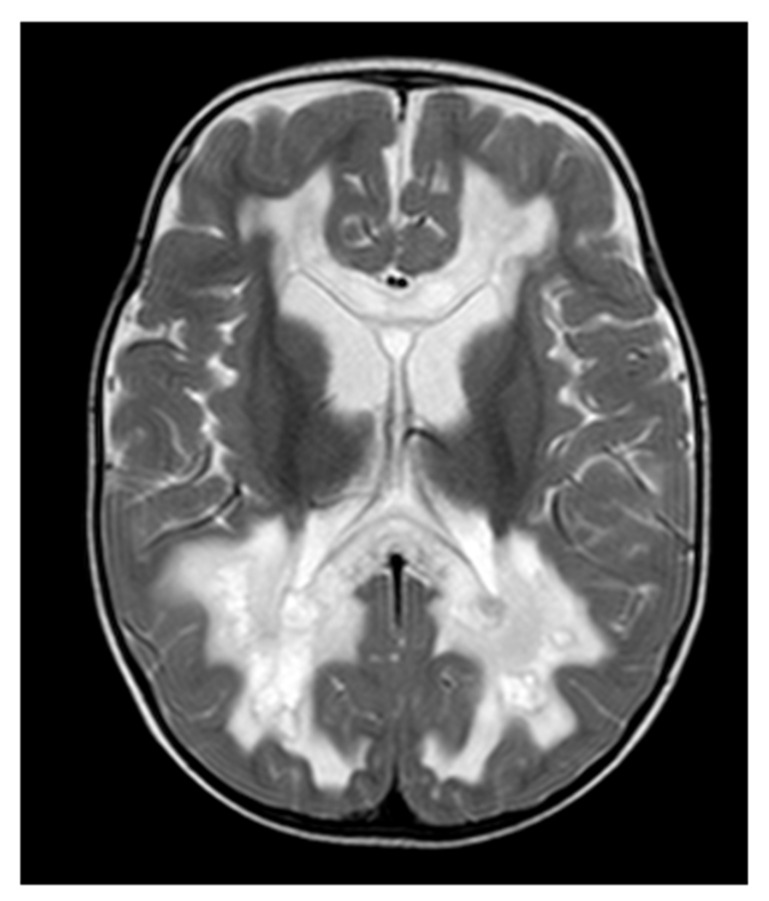
Neuroimaging in PDSS1 deficiency: Brain MRI (T2-weighted, axial view) of a 24-month-old boy with PSSD1 deficiency. Extensive leukoencephalopathy is visible with cystic white matter lesions in the occipital regions. Other MRI images of this individual were published previously [16].

In one patient clinically presenting with severe developmental delay, seizures, tremor, optic atrophy and hearing impairment, brain MRI at 12 months of age revealed mild cerebral atrophy. At 24 months of age, cystic white matter changes were found. In addition, there were signal abnormalities around the lateral ventricles and commensal fibers consistent with small necrotic or porencephalic cysts [16]. An MRI image of this individual is depicted in Figure 2. The cerebral MRI of another patient at the age of 6 months showed leukencepalopathy and a lactate peak on MR spectroscopy [15]. In the remaining other two patients, no brain abnormalities were found.

### 3.2. PDSS2 Deficiency

The *PDSS2* gene encodes decaprenyl diphosphate synthase subunit 2, which is required for the synthesis of the decaprenyl tail of CoQ_10_ and forming a heterotetramer with the *PDSS1* gene. Disease-causing *PDSS2* variants are also rare and are predominantly associated with nephropathology (primary coenzyme Q_10_ deficiency-3; COQ10D3; OMIM #614652). They were first described by Rötig et al. in 2000 [17]. 

Although almost all the seven published individuals showed neurological abnormalities, only one patient’s cerebral imaging was described in the literature [18,19,20]. Lopez et al. published a case in which a *PDSS2* variant caused Leigh syndrome. The patient showed muscular hypotonia and seizures amongst other non-neurological symptoms. The brain MRI, which was performed at the age of 5 months, showed bilateral symmetric abnormalities with elevated T2 and decreased T1 signal intensity in the basal ganglia [21].

### 3.3. COQ2 Deficiency 

*COQ2* encodes para-hydroxybenzoate (PHB)–polyprenyl transferase, which catalyzes the transfer of para-hydroxybenzoate to the polyprenyl chain. The first known patient was described by Quinzii et al. in 2005 [22]. Primary COQ2 deficiencies may present with a wide spectrum of disease severity (primary coenzyme Q_10_ deficiency-1; COQ10D1; OMIM #607426). Some affected individuals already deteriorate during the first months of life with severe encephalopathy, lactic acidosis and renal failure. Later-onset cases may manifest with steroid-resistant nephrotic syndrome and ataxia. Moreover, COQ2 deficiency is a known cause of multiple system atrophy (MSA) in late adulthood (OMIM #146500) [23]. 

Hashemi et al. reported a case in which one of two affected siblings showed cerebellar atrophy and white matter abnormalities on T2 and FLAIR brain MRI sequences [24]. In another individual, brain MRI at the age of four months revealed bilateral increased signal intensities in the putamen and cerebral cortex (Leigh pattern) [25]. Diomedi-Camassei et al. described a child with a normal brain MRI at the age of 22 months [26]. However, another individual from this study showed cortical and subcortical stroke-like lesions as well as diffuse cerebral atrophy at the age of 6 months. MR spectroscopy revealed a lactate peak within the affected brain regions [26]. The same study group reported an individual, who underwent brain MRI at the age of 33 months, which showed cerebellar as well as cerebral atrophy and stroke-like lesions [22]. 

### 3.4. COQ4 Deficiency

*COQ4* encodes a protein of the inner mitochondrial membrane that apparently has no enzymatic function but seems to be essential for the stabilization of the COQ multi-enzyme complex [27,28]. The clinical manifestation of COQ4 biosynthesis disorders was first described by Brea-Calvo et al. in 2015 (primary coenzyme Q_10_ deficiency-7; COQ10D7; OMIM #616276) [29]. Recently, our group and collaborators analyzed the imaging findings in a large cohort of individuals with COQ4 deficiency (44 individuals; 36 cases with extensive brain MRI studies) [30]. 

The most frequent finding was global cerebral atrophy. In addition, cerebellar abnormalities were found in the majority of cases. Cerebellar pathology included cases with hypoplasia (detected on pre- and early postnatal imaging) as well as cases with progressive atrophy. Another frequent abnormality was delayed myelination, which was seen in about half of the individuals. A unique/specific finding appeared to be a cystic malformation/degeneration of the cerebellum, which was observed in some of the individuals with severe and early disease onset. Another imaging finding in a subset of COQ4 patients were occipito-pariental stroke-like lesions. Apart from this MELAS-like pattern, individuals with a Leigh-like MRI pattern were also identified. 

Based on the analysis of brain MRIs in COQ4 patients, a phenotypic classification with three subtypes was proposed: Type 1: cerebral atrophy and a mixture of cerebellar atrophy and hypoplasia. Affected individuals showed a severe clinical phenotype. Type 2: stroke-like lesions and mild global brain atrophy. Patients presented with an intermediate clinical phenotype. Type 3: nonspecific changes with mild, generalized brain atrophy, slightly delayed myelination and, in some cases, even normal brain MRI. Affected individuals showed a late-onset phenotype. 

Adult-onset cases of COQ4 deficiency were reported only recently [31]. Three individuals presented with spastic paraparesis, whereas three other patients showed cerebellar ataxia. Brain MRI results were available in three individuals showing normal findings (one patient) as well as moderate cerebellar atrophy (two patients). 

**Figure 3:**
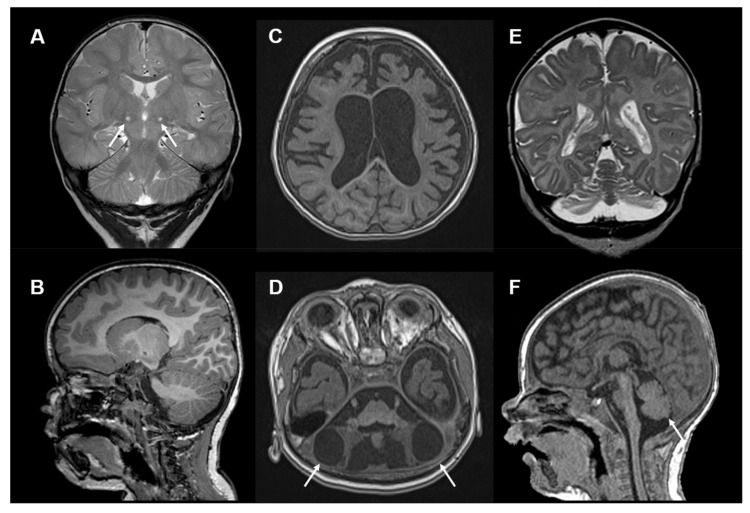
Neuroimaging in COQ4 deficiency: (**A**) Brain MRI, T2-weighted, coronal images of a 15-month-old girl with COQ4 deficiency. Images show bilateral circumscribed lesions in the subthalamic nuclei (white arrows). (**B**) Sagittal T1-weithed MRI images show no cerebellar lesions. (**C**) Brain MRI, T1-weighted, axial images of an 18-month-old girl with COQ4 deficiency showing global brain atrophy. (**D**) Brain MRI, T1-weighted, axial images of the same child showing cerebellar degeneration with bilateral large cysts within the cerebellar hemispheres (white arrows). (**E**,**F**) T2-weighted, coronal and sagittal images and of a 2-month-old girl with COQ4 deficiency showing cerebellar hypoplasia (white arrow). Other MRI images of these individuals were published previously [30].

Examples of brain MRI imaging findings in COQ4 deficiency are depicted in Figure 3. 

### 3.5. COQ5 Deficiency

*COQ5* encodes C-methyltransferase, which is responsible for C-methylation in the synthesis of CoQ_10_ [32]. Malicdan et al. were the first to report three siblings suffering from COQ5 deficiency in 2018 (coenzyme Q_10_ deficiency-9; COQ10D9; OMIM #619028) [33]. Brain MRI of one of the siblings at the ages of 8 and 16 years showed mild non-progressive cerebellar atrophy. The brain MRI of an older sister (age 22 years) revealed similar findings. Clinically the three sisters presented with neurological disorders, including cerebellar ataxia, epilepsy and intellectual disability. 

### 3.6. COQ6 Deficiency

*COQ6* encodes monooxygenase-6, which is responsible for catalyzing one or more ring hydroxylation steps [34]. Heeringa et al. first described individuals with COQ6 deficiency in 2011 [35]. COQ6 deficiencies mainly cause a renal phenotype that may be associated with deafness or visual impairment (primary coenzyme Q_10_ deficiency-6; COQ10D6; OMIM #614650). The clinical features show an overlap with PDSS1 and PDSS2 deficiency. Perrin et al. performed brain MRI in one of the individuals not showing any abnormalities [34]. Wang et al. reported an individual with COQ6 deficiency, who underwent brain MRI due to a seizure at the age of 5 months. The MRI showed a widening of the bilateral frontotemporal subarachnoid space and delayed myelination. 

The brother of this patient, also suffering COQ6 deficiency, received a computed tomography (CT) of the brain at the age of 5 months, which showed a similar pathology with signs of frontotemporal brain atrophy [36].

### 3.7. COQ7 Deficiency

The *COQ7* gene encodes for 5-demethoxyubiquinone hydroxylase—a protein in the inner mitochondrial membrane responsible for hydroxylation of 6-demethoxyubiquinone [37]. The first patient with a COQ7 deficiency was reported by Freyer et al. in 2015 (coenzyme Q_10_ deficiency-8; COQ10D8; OMIM #616733). Brain MRI was performed two months after birth, which showed no abnormalities [38]. 

In 2017, a patient of Wang et al. whose neurological development delay became noticeable in the second year of life had serial normal MRI scans of the brain and spine [39]. A second patient was described in 2022 by Wang et al. with global developmental delay at 15 months of age. The brain MRI showed hyperintensities in the supratentorial bilateral periventricular white matter on T2-weighted and FLAIR imaging [40].

Kwong et al. reported the case of a neonate with “encephalo-myo-nephro-cardiopathy”. Brain MRI at the age of 10 months showed multiple T2-hyperintense cystic changes involving bilateral corona radiata, basal ganglia and thalami as well as frontal cerebral atrophy. MR spectroscopy showed a lactate peak [41]. A nine-year-old girl with neurological symptoms and COQ7 deficiency referred to by Hashemi et al. showed no gross abnormalities in MRI of the brain and spine [24].

In December 2022, Jacquier et al. reported a case series of three grown-up siblings suffering from distal hereditary motor neuropathy due to disease-causing *COQ7* variants [37]. They performed brain MRI in one of the siblings showing no abnormalities. The study by Jacquier et al. illustrates that the clinical spectrum of COQ7 deficiency is much broader than previously thought and reaches up to adult-onset manifestations. This observation is comparable to recent findings in COQ4 deficiency (see above). 

**Figure 4:**
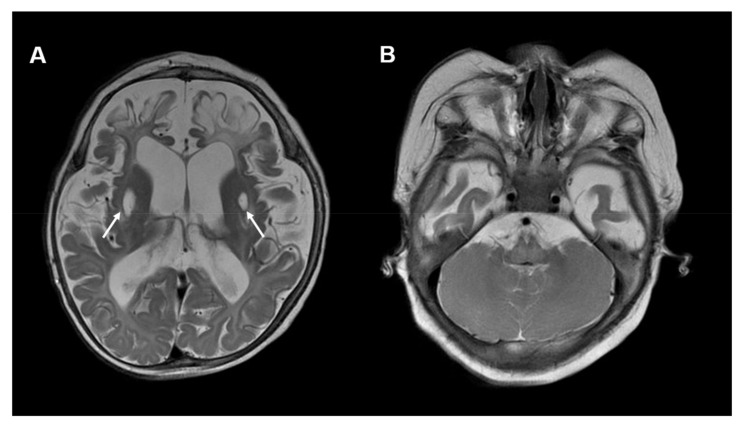
Neuroimaging in COQ7 deficiency: (**A**) Brain MRI, T2-weighted, axial images of a 10-month-old boy with COQ7 deficiency. The MRI shows global brain atrophy and areas of encephalomalacia in bilateral frontal lobes. In addition, symmetric cystic changes within the putamen are visible (white arrows). (**B**) No cerebellar abnormalities are visible. Other MRI images of this individual were published previously [41].

An example of imaging findings in COQ7 deficiency is depicted in Figure 4. 

### 3.8. COQ8A Deficiency

**Figure 5:**
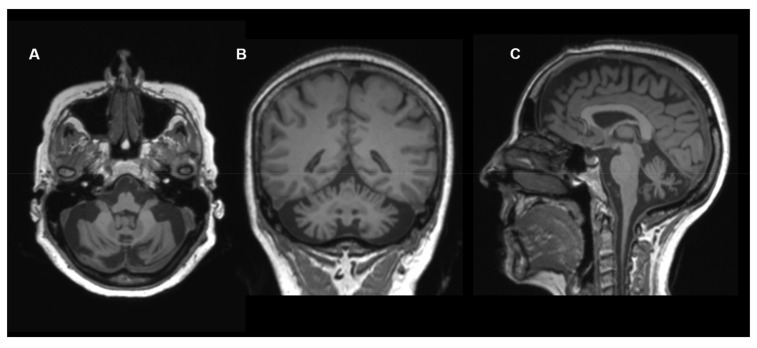
Neuroimaging in COQ8A deficiency: Brain MRI (T1-weighted images, (**A**) axial view; (**B**) coronal view; (**C**) sagittal view) of a 60-year-old female with COQ8A deficiency. Images show cerebellar atrophy. Other MRI images of this individual were published previously [45].

*COQ8A* encodes for an atypical kinase-like protein relevant for CoQ_10_ biosynthesis [42,43,44]. Its exact function is still unclear. Disease-causing variants in *COQ8A* were first described in 2008 (primary coenzyme Q_10_ deficiency-4; COQ10D4; OMIM #612016) [44]. In 2020, Traschütz et al. published a large cohort of 59 COQ8A individuals, 54 of whom had received brain MRI [45]. They identified cerebellar atrophy as an almost universal finding (94% of patients). Less frequently, cerebral atrophy (8%), stroke-like abnormalities (8%), infratentorial signal abnormalities (4%) and brainstem atrophy (2%) were observed. No structural basal ganglia abnormalities were found. Standardized DTI in three individuals revealed changes of the infratentorial fiber tracts [45]. The findings were consistent with previous reports [44,46]. An example of cerebellar atrophy caused by COQ8A deficiency is depicted in Figure 5. 

In 2020, Zhang et al. reported a 35-year-old patient who suffered neurological symptoms, such as early-onset exercise intolerance and progressive cerebellar ataxia, wide-based gait and tremors, accompanied by symptoms of dysautonomia since he was nine years old. The brain MRI showed cerebral atrophy [43].

In 2022, a 11-year-old girl with persistent seizures and developmental delay was reported by Ashrafi et al. [47]. She had brain MRI at 2 and 3 years of age, which revealed mild cerebellar atrophy and stroke-like signal changes. Multifocal cortical involvement and cerebellar atrophy were shown at 5 and 7 years of age. At the age of 11 years, brain MRI revealed progressive cerebellar atrophy and stroke-like cortical involvement.

That same year, a 16-year-old girl presented to Degerliyut et al. with recurrent seizures and ataxia [48]. Brain MRI revealed severe cerebellar atrophy, stroke-like lesions and a lactate peak on MR spectroscopy.

In 2022, a case report by Paprocka et al. described a 22-month-old girl, who showed signs of regression. MRI of the brain and cervical spine showed no abnormalities [49]. The follow-up MRI at the age of 2.5 years still did not show any signs of cerebellar atrophy.

### 3.9. COQ8B Deficiency

*COQ8B* is a paralog of *COQ8A* and also encodes for an atypical kinase with unclear function [50]. *COQ8B* variants mainly cause a phenotype with kidney damage and nephrotic syndrome (nephrotic syndrome type 9; NPHS9; OMIM #615573). The disease was first described by Ashraf et al. in 2013 [51]. Zhai et al. performed a brain MRI in a 3-year-old child to rule out a potential neurological involvement. The brain MRI showed no abnormalities [52]. Thus far, no further cases with cranial imaging have been reported in the literature.

### 3.10. COQ9 Deficiency

*COQ9* encodes a lipid-binding protein that associates and functions in combination with COQ7 [53]. Rahman et al. and Duncan et al. first reported on a neonate who, at 6 h of age, presented with poor feeding, hypothermia, increased muscle tone and lactic acidosis in 2001/2009 (primary coenzyme Q_10_ deficiency-5; COQ10D5; OMIM #614654) [54]. The child later developed seizures and presented with severe global developmental delay. Brain MRI showed cerebral and cerebellar atrophy [54,55]. Furthermore, Danhauser et al. described a patient who developed respiratory failure, muscular hypotonia and seizures as well as elevated lactate levels in the blood during the neonatal period. A brain ultrasound showed multiple choroid plexus cysts and symmetrical signal alterations in the basal ganglia, consistent with neonatal Leigh syndrome [56]. 

In 2018, Smith et al. reported a family with four siblings suffering from COQ9 deficiency [57]. The first sibling showed prenatal abnormalities with intrauterine growth restriction (IUGR). The child was born premature and showed mild hydrocephalus and lobulated cysts in the frontal lobes on brain ultrasound. Neurological examination was abnormal with central vision loss and an abnormal movement pattern. Furthermore, the child had severe lactic acidosis. In the second sibling, the pregnancy was terminated due to IUGR, cystic kidney changes and oligohydramnios. 

Multifocal global ischemic events in the brain were identified postmortem. The third child born to these parents also suffered from IUGR. At 33 weeks gestation, on fetal MRI, a cranial hemorrhage in the left lateral ventricle was suspected. After birth, the child presented with lactic acidosis and died during the first day of life. Brain autopsy showed a Leigh-syndrome pattern as well as a subventricular cystic degeneration around the lateral ventricles. The fourth child of the family presented with similar pathologies. However, a postmortem brain autopsy did not show clear abnormalities. 

In 2019, Olgac et al. reported on a 9-month-old female infant with growth retardation, microcephaly and seizures. Brain MRI revealed hypoplasia of the cerebellar vermis and brain stem, corpus callosum agenesis and cortical atrophy [58].

### 3.11. HPDL Deficiency

*HPDL* encodes 4-hydroxyphenylpyruvate dioxygenase-like protein, whose function is not fully understood by now [59]. Recent studies indicate that 4-hydroxymandelate (4-HMA), an intermediate involved in CoQ_10_ biosynthesis, is a product of HPDL [6]. In 2020, Husain et al. described clinical and brain MRI findings in 17 individuals with bi-allelic *HPDL* variants (OMIM #619026) [60]. Clinical features in these patients reached from developmental delay, seizures and microcephaly to non-neurological manifestations. Based on the MRI images, the authors described different patterns of brain lesions. Abnormalities included symmetrical T2-hyperintensities of basal ganglia and brain stem, white matter abnormalities and cortical lesions. In one individual, there was suspicion of a transient spinal pathology. Three out of four individuals showed a lactate peak on MR spectroscopy. 

In 2021, Wiessner et al. reported 34 individuals with HPDL deficiency. Phenotypically, all individuals described showed spasticity (OMIM #619027) [61]. Some of the individuals presented with ataxia or oculomotor abnormalities. The most severely affected patients showed global developmental delay, seizures and encephalopathy. Depending on the severity of the clinical phenotype, patients were divided into three groups. Individuals showing the mild phenotype displayed no abnormalities on brain MRI. Patients with intermediate and severe phenotypes presented with corpus callosum abnormalities. Cerebellar atrophy and bilateral inferior olivary hyperintensities on T2-weighted MRI images were shown for the intermediate phenotype. One individual with a severe phenotype presented with an MRI consistent with Leigh syndrome, and MR spectroscopy showed a lactate peak [62].

In 2022, Wang et al. identified *HPDL* variants in a 6-month-old male infant. Clinically the boy presented with developmental delay, seizures and spasticity. Brain MRI revealed a thin corpus callosum, ventriculomegaly and white matter volume reduction [63].

In 2022, Micule et al. described two individuals with disease-causing *HPDL* variants [64]. One child presented with muscular hypertonia, seizures and regression at the age of 6 weeks. The brain MRI showed white matter abnormalities. No lactate peak was seen on MR spectroscopy. In the second child, brain MRI at 5 weeks of age revealed significant diffuse white matter abnormalities, sparing basal ganglia and a lactate peak on MR spectroscopy. At the age of 2 years, follow-up MRI showed severe cortico-subcortical atrophy and white matter abnormalities.

**Figure 6:**
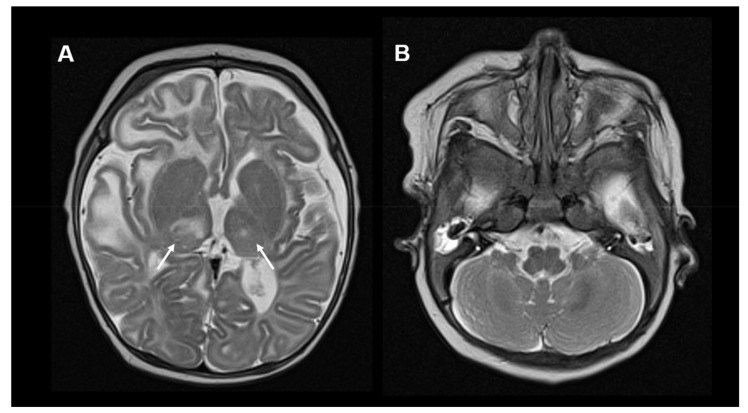
Neuroimaging in HPDL deficiency: (**A**) Brain MRI, T2-weighted images of a 3-month-old girl with HPDL deficiency. Images show subcortical T2-hyperintensities, mainly affecting the right frontotemporal regions. Moreover, asymmetrical signal abnormalities of the thalami are visible (white arrows). (**B**) No cerebellar abnormalities are visible.

In addition to the reported individuals, we recently identified an infant with HPDL deficiency (not yet published). The girl presented with seizures, global developmental delay and microcephaly at the age of 3 months. The brain MRI showed subcortical T2-hyperintense lesions predominantly in the right frontal region (see Figure 6). Moreover, bilateral thalamic lesions were seen. MR spectroscopy did not reveal clear abnormalities.

## 4. Discussion

Neuronal damage and progressive neurodegeneration are hallmarks of mitochondrial diseases. The findings summarized here clearly indicate that this also holds true for primary CoQ_10_ deficiency disorders. Brain MRI abnormalities observed in affected individuals include cerebral and cerebellar atrophy as well as the classical Leigh-syndrome pattern with symmetrical lesions of the basal ganglia and brain stem. Moreover, several CoQ_10_ deficiency disorders may present with metabolic strokes, comparable to lesions observed in MELAS syndrome (mitochondrial encephalomyopathy with lactic acidosis and stroke-like episodes). In addition, abnormalities of the white matter and disturbed myelination are a frequently reported finding. 

The rarity of primary CoQ_10_ deficiency disorders makes it difficult to capture the full clinical spectrum of the different genetic defects. For human COQ2 deficiency, which was first described almost 20 years ago [22], it is known that disease manifestations range from prenatal abnormalities (oligohydramnios, intrauterine growth restriction, cerebellar hypoplasia [57,65]) and neonatal encephalopathy up to neurodegeneration in late adulthood (multiple system atrophy; MSA). 

Recent larger studies on COQ4 deficiency show a comparable disease range [30]. For COQ8A deficiency, a broad clinical spectrum has been reported, and even for the extremely rare COQ7 deficiency, research demonstrated that late-onset disease forms with hereditary motor neuropathy are possible [37]. These findings suggests that the majority of CoQ_10_ deficiency disorders share a common spectrum of neuropathology. Interestingly, also retinal affection seems to be a rare disease feature of many COQ defects (e.g., PDSS1, COQ2, COQ4 and COQ5) [66]. 

In 2022, hydroxyphenylpyruvate dioxygenase-like (HPDL) protein was identified to play a role in CoQ_10_ biosynthesis [6]. HPDL deficiency was first described in 2020 [60]. In line with the idea that HPDL is a CoQ_10_ biosynthesis enzyme, the clinical manifestation of HPDL deficiency ranges from early-onset encephalopathy up to adolescent-onset spastic paraplegia. This phenotypic spectrum partially overlaps with that of human COQ4 deficiency. 

Regarding brain pathology, the cerebellum appears to be particularly vulnerable in individuals with certain CoQ_10_ deficiency disorders, leading to cerebellar atrophy and ataxia. This is a typical finding in adult-onset forms of COQ2, COQ4 and COQ8A deficiency. However, also the early development of the cerebellum may be disturbed. Cerebellar hypoplasia and cerebellar cysts are hallmarks of severe COQ4 defects [30]. However, cerebellar hypoplasia was also reported in one child with COQ9 deficiency. Interestingly, Pdss2 knockout in mice resulted in cerebellar hypoplasia and in the development of cerebellar ataxia [67]. In humans, the neuropathology of PDSS2 defects has not been investigated in detail so far. 

Apart from cerebellar hypoplasia, the development of the corpus callosum may also be disturbed in certain CoQ_10_ biosynthesis disorders. This especially holds true for HPDL deficiency with several reported cases but was also described in COQ9 deficiency. 

As mentioned above, stroke-like episodes were reported as a potential disease feature of several CoQ_10_ biosynthesis defects (COQ2, COQ4 and COQ8A) [30,45,48,68,69]. Clinically, affected individuals may present with focal seizures, altered consciousness and hemiparesis. The underlying pathophysiology is not fully understood. Focal vasogenic edema and neuronal hyperexcitability are key aspects [70]. Importantly, stroke-like lesions need to be distinguished from ischemic stroke and are not confined to an arterial territory. In classical MELAS syndrome, the temporal and occipital brain regions are most frequently affected. For CoQ_10_-deficiency disorders, no detailed studies on the pattern of brain lesions during stroke-like episodes are available thus far. 

Another brain pathology observed in individuals with COQ defects is disturbed myelination and leukoencephalopathy (PDSS1, COQ2, COQ6, COQ7 and HPDL). The importance of CoQ_10_ in myelination is supported by several research studies. Hyung et al. demonstrated that CoQ_10_ treatment markedly facilitated myelination in a cell-culture system using mouse motor neurons and Schwann cells [71]. 

Moreover, Ota et al. (2014) demonstrated an altered subcellular localization of 25α/tubulin polymerization-promoting protein (TPPP) in oligodendrocytes of an individual with multiple system atrophy due homozygous variants in *COQ2*. TPPP plays a crucial role in myelination and in the stabilization of microtubules [72]. Interestingly, genetic defects of mitochondrial complex II (succinate dehydrogenase) and mitochondrial complex III (cytochrome bc1 complex) are frequently associated with leukoencephalopathy [73,74]. In this context, it is important to know that the activity of complex II/III and the levels of CoQ_10_ are correlated, which might play a role in these clinical similarities [75]. 

Of note, certain CoQ_10_ biosynthesis defects predominantly affect the kidney (PDSS2 and COQ6), and so far, no major effects on the nervous system have been described. There is currently no explanation for these phenotypic differences. A tissue-specific effect of the different COQ proteins might be the underlying cause. Interestingly, in yeast, it was shown that COQ proteins, which modify the quinone head, assemble into resolvable domains in vivo, representing COQ complexes. 

These protein complexes are essential for CoQ production and mitochondrial function [76]. This phenomenon might also play a role in the cell-type/tissue-specific effects of COQ defects. For a better understanding of the CoQ_10_ metabolism in vivo, it is desirable to develop and apply novel brain imaging strategies beyond classical MRI. Positron emission tomography (PET) imaging of ^11^C-labeled CoQ_10_ was applied in a rat model to monitor the time course of CoQ accumulation in different organ systems [77]. However, no applications in humans have been reported so far. For monitoring of cerebral bioenergetics, metabolic ^31^phosphorus magnetic resonance spectroscopy imaging (^31^P-MRSI) was applied in two individuals with COQ8A deficiency [78]. 

This technique allows the detection of ATP production and the quantification of phosphocreatine levels. In COQ8A deficiency, ^31^P-MRSI was used to access the effects of CoQ_10_ supplementation on the cerebellar bioenergetic state. This might be an interesting marker to access the response to CoQ_10_ supplementation and to guide treatment decisions. Importantly, new techniques to monitor treatment responses gain additional importance in view of novel metabolic bypass strategies for certain CoQ_10_ biosynthesis disorders, which were developed in cell culture as well as animal models and which are close to applications in humans [38,79,80,81,82,83,84]. 

## 5. Conclusions

There have been major advances in our understanding of the pathophysiology of primary CoQ_10_ biosynthesis disorders during recent years. Numerous novel disease genes have been identified. However, our knowledge regarding the phenotypic spectrum of these disorders is still incomplete. The neuropathology of CoQ_10_ biosynthesis disorders shows a broad overlap with other mitochondrial diseases, which makes it very difficult to distinguish these entities on the basis of neuroimaging. Nevertheless, certain findings (e.g., cerebellar cysts in COQ4 deficiency) may provide specific diagnostic clues. 

COQ defects may present even before birth (e.g., cerebellar hypoplasia and dysplasia of the corpus callosum) with a continuous diseases spectrum up to late adulthood. In view of the potential treatment option with CoQ_10_ supplementation, early diagnosis and tools for treatment monitoring are warranted. However, the appropriate dosage, formulation and biodistribution of CoQ_10_ are still a matter of debate. In particular, the efficiency of CoQ_10_ uptake after oral supplementation into the central nervous system is still unclear. In this context, additional diagnostic strategies, such as ^31^P-MRSI or PET imaging of labeled CoQ_10_, might provide novel options to guide treatment.
